# Adolescent presentation of L-type ccTGA with pulmonary valve hypoplasia and RV dysfunction: from cardiogenic shock to surgical repair

**DOI:** 10.1186/s43044-026-00733-8

**Published:** 2026-04-01

**Authors:** Shubham Jain, Deepak Kumar, Naween Kumar, Pankaj Mohan Shrivastava

**Affiliations:** Darbhanga Medical College and Hospital, Darbhanga, India

## Abstract

**Background:**

Congenitally corrected transposition of the great arteries (ccTGA) is an uncommon congenital heart defect involving both atrioventricular (AV) and ventriculoarterial (VA) discordance. This condition is further complicated by additional anomalies such as a hypoplastic pulmonary valve, ventricular septal defect (VSD), and dextroversion. These abnormalities may contribute to an increased risk of heart failure and cardiogenic shock, making early detection and appropriate intervention essential for better patient outcomes.

**Case presentation:**

A 16-year-old female presented with progressive shortness of breath, cyanosis, recurrent episodes of syncope, and exertional palpitations. Upon admission, the patient exhibited severe hypoxia, tachycardia, and hypotension, consistent with cardiogenic shock. She was NYHA grade IV at the time of presentation.Imaging studies, including chest X-ray and echocardiography, GLS- strain imaging and cardiac MRI confirmed the diagnosis of ccTGA with AV and VA discordance, a left-sided aortic arch, subaortic VSD, a hypoplastic pulmonary valve, and left sided morphologic right ventricular systolic dysfunction. Blood investigations indicated polycythemia and signs of congestive hepatopathy.

**Management and outcome:**

The patient was initially stabilized with inotropic support using norepinephrine (0.05–0.5 mcg/kg/min), along with diuretics and ACE inhibitors. Over the course of seven days, her condition improved significantly, allowing for the withdrawal of inotropic support. She was subsequently referred for surgical evaluation, with potential options including the double-switch procedure or VSD closure, depending on the extent of structural and functional abnormalities. Our patient underwent double switch procedure despite having RV dysfunction and improved significantly over a period of 3 months.

**Conclusion:**

This case underscores the significance of prompt recognition and aggressive management in complex congenital heart conditions like ccTGA. Cases of symptomatic ccTGA in adolescence are exceptionally rare, highlighting the importance of a multidisciplinary approach in optimizing patient care and surgical planning.

## Introduction

A rare congenital cardiac anomaly, congenitally corrected transposition of the great arteries (ccTGA) of L type and its association with a hypoplastic pulmonary valve is relatively uncommon. In this condition, the atria are connected to the opposite ventricles, that is the left sided morphologic right ventricle receives blood from the left atrium, and the right sided morphologic left ventricle receives blood from the right atrium, which is known by the term atrioventricular (AV) discordance. Additionally, the ventricles are reversed and connected to the opposite great arteries, i.e. the aorta receives blood from the left sided morphologic right ventricle, and the pulmonary artery receives blood from the right sided morphologic left ventricle, which is known as ventriculoarterial (VA) discordance. Another variation seen with ccTGA is dextroversion in which the heart is positioned to the right side. In ccTGA, the aorta is typically positioned antecedently and lies on the left side of the pulmonary artery, which is characteristic of L-transposition. Despite these anatomical anomalies, systemic circulation remains physiologically corrected, hence the term “congenitally corrected” transposition [1–3]. 

Other conditions that are often associated with this congenital cardiac anomaly are ventricular septal defects (VSDs) and valve abnormalities of the pulmonary valve, including atresia. Over time, patients may experience complications like ventricular dysfunction, arrhythmias (e.g., ventricular fibrillation), with or without complete heart block, and heart failure, which can be life-threatening at times. Factors such as advancing age, strenuous physical activity, and pregnancy can exacerbate these issues.

An emerging surgical intervention for ccTGA is the double-switch procedure. Apart from this, in patients without pulmonary valve atresia, this approach involves an atrial baffle operation to redirect systemic and pulmonary venous return, followed by repositioning of great artery connections known as arterial switch procedure, thereby restoring normal physiological circulation.

## Case summary

A 16-year-old female patient of low socioeconomic status presented to the medicine emergency department with complaints of breathlessness, cough with purulent sputum.

The patient developed shortness of breath, which was present even at rest (NYHA grade IV), retrosternal chest pain, which is a pinprick type of pain that is non- radiating and aggravates with exertion; along with dizziness and palpitations that aggravate with exertion for 6 months.

The patient had cyanosis, which was insidious in onset and suffered 4–5 episodes of syncope within the last 6 months.

She had history of recurrent coughs and colds since childhood.

The patient has no history suggestive of paroxysmal nocturnal dyspnea, orthopnea, hemoptysis, hematemesis, black stools, joint pain, and rash.

There was no family history suggestive of sudden cardiac death (SCD) in the last 4 generations and consanguineous marriage in the family.

### Past interventions

Despite having recurrent respiratory symptoms since childhood along with syncopal episodes in the past, the patient had never undergone proper cardiac imaging or consultation with a physician or cardiologist. This delay was mainly due to lack of awareness and financial constraints.

### Clinical findings

Upon admission, the patient exhibited breathlessness, cyanosis, and tachypnea. The patient also had a high-grade fever and was hemodynamically unstable with a weak thready pulse having a pulse rate of 140 beats per minute, measured blood pressure of 72/40 mm of Hg, having tachypnea with a respiratory rate of 26 per minute. Oxygen saturation (SpO₂) was found to be critically low at 47% on room air.

On physical examination, central cyanosis, digital clubbing (grade 3), and pedal edema (grade 2) were noted.

She was conscious and irritable.

Bilateral lung air entry was reduced likely due to pulmonary congestion and basal fine crepitations in both lung fields.

Auscultation findings: Cardiac examination revealed tachycardia along with a grade IV ejection systolic murmur (ESM) heard prominently over the pulmonary area.

The patient’s abdomen was soft and non-distended, and the liver was just palpable below the right hypochondrium.

Acute chest infection as supported by patient’s symptoms and investigations is the likely cause of her acute decompensation.

**Table:** Timeline of events

Step	Days
Day 0	Presented with shock and cyanosis → Started on noradrenaline and IV diuretics
Day 1–3	Stabilization of vitals, improved saturation
Day 4–5	Weaned off inotropes, oral ACE inhibitors and beta-blockers started
Day 7	Clinical improvement, referred for surgical evaluation (not associated with blood), and fever for 10 days.
Day 16	Patient underwent surgical intervention.
3 months	Followed up for Post op improvement

## Diagnostic assessment

During admission, ECG with leads on left side of chest had sinus tachycardia, right axis deviation (around 100 degree), left sided morphologic right ventricular hypertrophy with strain pattern and T wave inversion in leads V1-3.


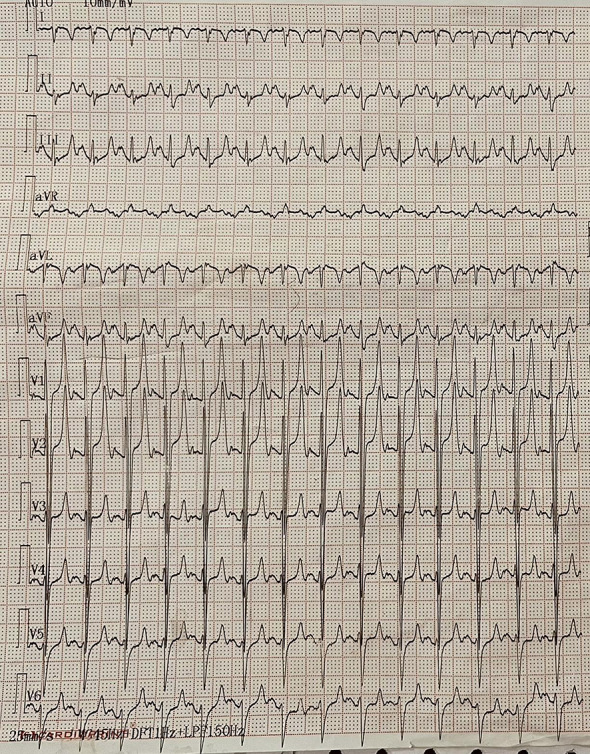


Digital chest x-ray revealed dextroversion, cardiomegaly possibly due to RVH, along with pulmonary vessel prominence to the right side and aortic prominence to the left side.


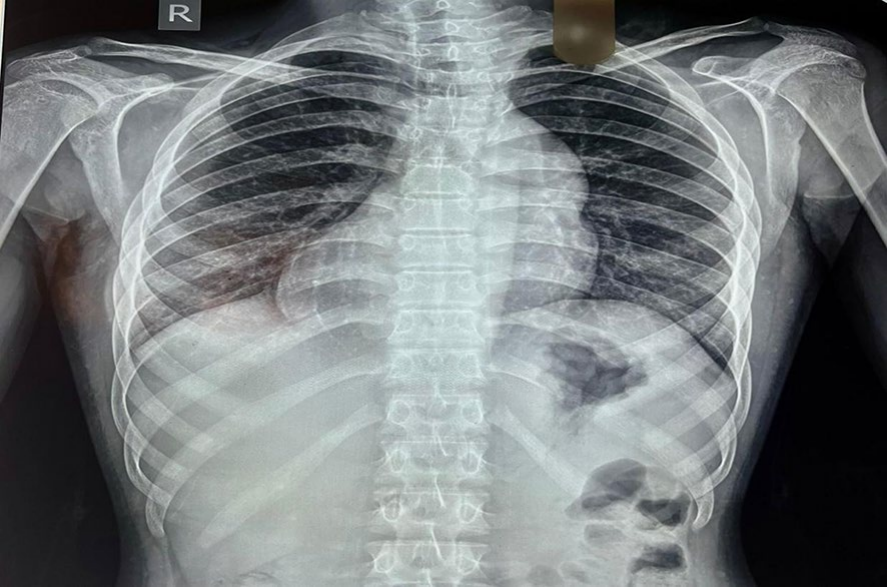


Echocardiography findings are

1. Situs solitus, dextroversion.
2. AV discordance.
3. VA discordance.
4. The aorta lies anterior to the pulmonary artery.
5. Minimal flow seen via the pulmonary valve (hypoplastic pulmonary valve).
6. Left sided aortic arch.
7. Subaortic VSD with flow from the right sided morphologic LV to the left sided morphologic RV.
8. left sided morphologic right ventricular systolic dysfunction is present with global hypokinesia seen in the left sided morphologic right ventricle.
9. Right sided morphologic left ventricular systolic function was normal with EF-60%.

Dimensions

**Table:** 

Left sided morphologic RV end diastolic area	27 cm^2^
Left sided morphologic RV basal diameter (PLAX)	48 mm
Left sided morphologic RV Fractional area change	22%
TAPSE	15 mm
Pulmonary valve annulus Z-score	–3.2 (hypoplastic)
Peak gradient across PV	65 mmHg
McGoon index	≈ 1.3

### Strain imaging

GLS (Global longitudinal strain) of

1. Systemic left sided morphologic RV is -13.5% which is suggestive of impaired RV longitudinal contractility.
2. Pulmonary right sided morphologic LV is -20% - normal range.
3. Regional strain abnormalities of basal and mid free wall segments of RV.

Cardiac MRI revealed.

1. ccTGA (L-looped ventricles, atrioventricular & ventriculoarterial discordance).
2. Subaortic VSD.
3. Hypoplastic pulmonary valve.
4. Dextroversion.
5. left sided morphologic right ventricular dysfunction.
6. Main pulmonary artery (MPA) diameter: 14 mm.
7. Right pulmonary artery (RPA) diameter: 7 mm.
8. Left pulmonary artery (LPA) diameter: 8 mm.
9. Systemic (left-sided morphologic RV):

- Indexed end-diastolic volume (EDV): 150 ml/m².
- Ejection fraction (EF): 30%.

10. Subpulmonic (right-sided morphologic LV):

- Indexed EDV: 70 ml/m².
- EF: 55–60%

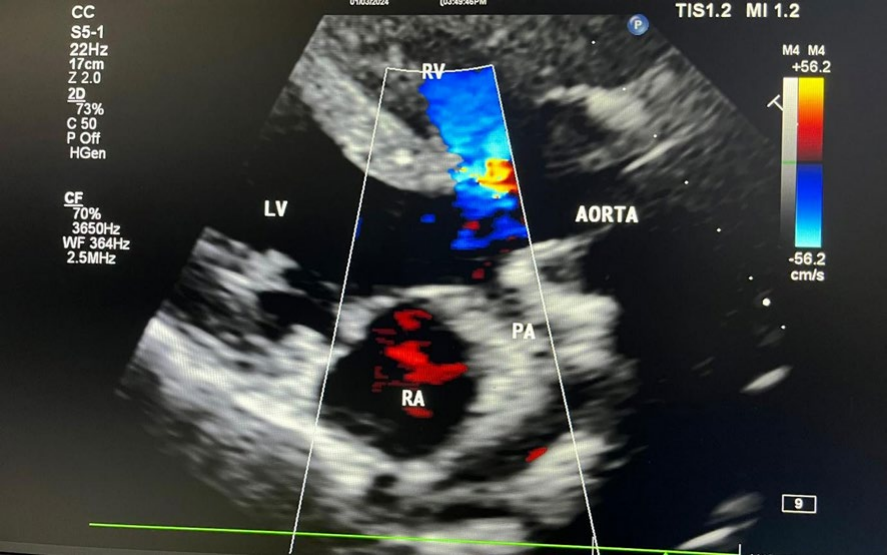



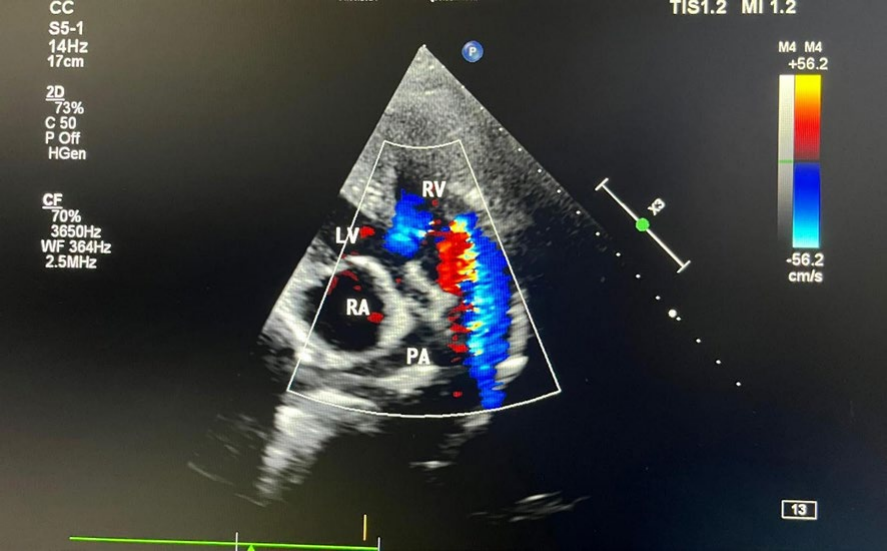



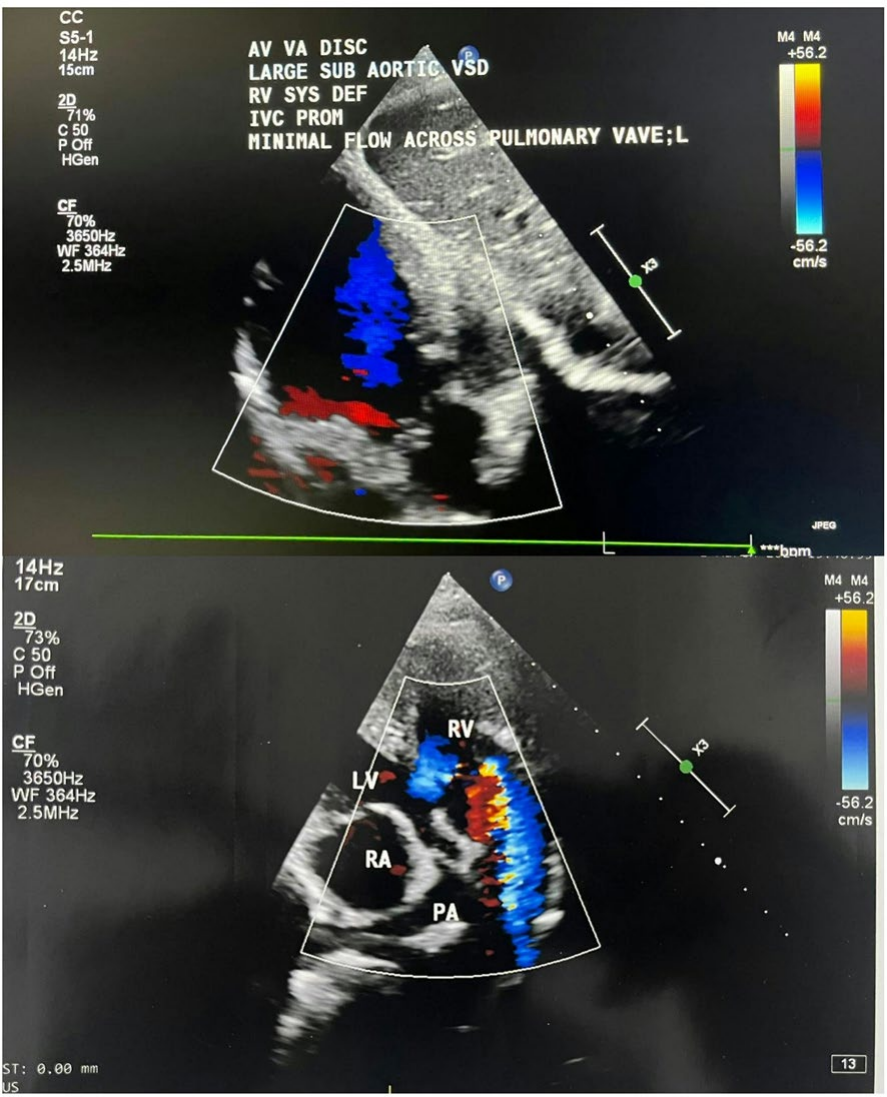


### Laboratory findings

The patient’s complete blood count revealed the following:

- Hemoglobin: Elevated at 21.7 g/dL.
- Mean Corpuscular Volume (MCV): Within normal range at 83.8 fL.
- Hematocrit: Increased to 75.9%.
- Total Leukocyte Count: 9,080/µL, with neutrophils comprising 80.1%.
- Platelet Count: 158,000/µL.

These findings indicate polycythemia, as evidenced by the elevated hemoglobin and hematocrit levels. The leukocyte and platelet counts are within normal limits.

Liver enzymes (AST and ALT) were elevated but < 2 ULN and ALP, total bilirubin levels are only mildly elevated which is suggestive of congestive hepatopathy. Blood urea nitrogen, sr. Creatinine, and random blood sugar levels seem normal. CRP levels were markedly raised, 132.63 mg/l.

### Diagnostic challenges

The complex nature of these anatomical anomalies and the rarity of ccTGA made early diagnosis difficult. Due to financial constraints and specialized cardiology services delayed detailed anatomical delineation.

## Diagnosis

The patient is having L-type congenitally corrected transposition of the great arteries (ccTGA), distinct by presence of AV and VA discordance. This complex cardiac anomaly is accompanied by dextroversion, pulmonary valve hypoplasia, subaortic ventricular septal defect (VSD), and left sided morphologic RV systolic dysfunction with heart failure and cardiogenic shock.

### Prognosis

The prognosis in our case with L type ccTGA is highly variable and depends on multiple factors. Early surgical intervention with proper follow up could substantially improve future outcomes. However, left sided morphologic right ventricular dysfunction along with pulmonary valve hypoplasia is having risk of heart failure and arrhythmias which is life threatening.

## Management/therapeutic intervention

At the time of the presentation, the patient had acute heart failure with cardiogenic shock.

Patient was started on inotropic support therapy with noradrenaline at the initial dose of 0.05 mcg/kg/min IV infusion followed by the maintenance dose of 0.5 mcg/kg/min.

Under coverage of inotropic support, inj. Frusemide 20 mg IV stat was given, followed by the maintenance dose of 10 mg thrice a day.

Patient improved clinically and hemodynamically within a span of 3 days.

After which, inotropic support was withdrawn and the lowest possible dose of ACE inhibitor/ARB along with Beta blocker were started.

Patient’s showed marked clinical improvement within a period of 7 days and was referred to CTVS for further treatment.

Outcome: The patient tolerated all prescribed medications without any reported side effects during hospitalization.

In general, management of L-type ccTGA with a hypoplastic pulmonary valve, VSD and dextroversion involves both medical and surgical strategies.

Rationale: Noradrenaline was used for initial hemodynamic support. Diuretics relieved volume overload. Beta-blockers were added to improve right ventricular function and prevent arrhythmias. ACE inhibitors reduced afterload.

### Medical management

Pharmacological therapy aims to optimize ventricular function and mitigate symptoms. Angiotensin II receptor blockers (ARBs) and angiotensin-converting enzyme (ACE) inhibitors are utilized to decrease right ventricular afterload and slow cardiac remodeling, thereby enhancing cardiac performance. Beta-blockers are also employed to manage arrhythmias and reduce myocardial oxygen demand.

### Surgical management

Surgical intervention remains the definitive treatment for ccTGA, with the approach determined by the age of presentation and the intensity of blood flow through the anatomical defects.

In patients with ccTGA and associated pulmonary outflow tract hypoplasia, palliative shunt operations such as a Blalock–Taussig (BT) shunt or bidirectional Glenn (BDG) may be considered, depending on the size of the pulmonary arteries and overall surgical feasibility. In our case, the pulmonary artery dimensions (MPA 14 mm, McGoon index ≈ 1.3) were considered adequate for proceeding with a double-switch procedure, obviating the need for a shunt-based palliation.

Available procedures are

- VSD closure alone.
- Double-Switch Procedure.

**Table:** 

Procedure	Goal	Remarks
VSD closure alone	Leave systemic left sided morphologic right ventricular in place; close defect to reduce volume overload	patch closure of the septal defect can be performed to block left-to-right shunting and preserve ventricular function.
Double-Switch Procedure	Redirect systemic circulation to morphologic LV (via arterial + atrial switch)	For patients with more complex anatomy, the double- switch operation is considered the gold standard. This approach includes an atrial baffle procedure to divert pulmonary vasculature blood to the right sided morphologic left ventricle and systemic vasculature blood to the left sided morphologic right ventricle. Subsequently, an arterial switch is performed to correct the ventriculoarterial discordance, thereby restoring normal physiological circulation and alleviating symptoms [4].

**Table:** 

Clinical parameter	Double-switch favored when…	VSD closure favored when…
Left sided morphologic right ventricular Function	Preserved early (can delay dysfunction)	Already mildly to moderately impaired
Right sided morphologic left ventricular dysfunction Preparedness (Training)	Right sided morphologic LV can handle systemic pressures (often needs training)	LV underdeveloped, unsuitable for systemic circulation
Presence of Pulmonary Stenosis or Hypoplasia	Helps LV training (↑ afterload)	If severe obstruction → may be addressed separately
Patient Age	< 12–15 yrs preferred for switch	Older patients with fixed RV dysfunction
Surgical Risk/Complexity	Acceptable in specialized centers	High risk or resource-limited centers

## Factors deciding type of procedure

After being stabilized, the patient was referred to a cardiothoracic unit to higher center.

Despite the presence of left sided morphologic right ventricular dysfunction, the surgical team opted for double-switch procedure given the relatively preserved LV function, patient’s young age, and feasibility of retraining systemic circulation early.

The decision to proceed with a double-switch operation, despite the presence of a hypoplastic pulmonary valve, was based on multidisciplinary consensus. Typically, a hypoplastic pulmonary valve would not be considered suitable as the neo aortic valve, as it may not sustain systemic pressures. However, in this case, the annular dimensions, pulmonary artery size, and surgical feasibility allowed the team to proceed with modifications. This underscores the importance of individualized surgical planning in ccTGA.

## Follow-up plan

Post-operative echocardiography confirmed successful anatomical correction with improved RV strain and clinical status at 3-month follow-up. Patient’s clinical status improved significantly from NYHA IV to NYHA ll and markedly improvement in SpO2 from 47% to 91% on room air during follow up.

Now Patient is under follow up, with monitoring of her vitals monthly and periodic assessment with echocardiography and strain imaging every 3 monthly.

**Table:** Comparison of pre- and post-operative imaging

Parameter	Pre-operative (Echo/CMR)	Post-operative (3 months, Echo/CMR)	Comments
Pulmonary valve annulus Z-score	–3.2 (hypoplastic)	Stable (not relevant post-op)	Contraindication for arterial switch, but double switch feasible
Main pulmonary artery (MPA) diameter	14 mm	15 mm	Mild growth
Right pulmonary artery (RPA) diameter	7 mm	8 mm	Improved flow
Left pulmonary artery (LPA) diameter	8 mm	9 mm	Improved flow
McGoon index	≈ 1.3	≈ 1.6	Suggests improved pulmonary circulation
Peak gradient across PV	65 mmHg	30 mmHg	Marked reduction
Systemic (left-sided morphologic RV) – Indexed EDV	150 ml/m²	120 ml/m²	Decreased dilation
Left-sided morphologic RV EF	30%	42%	Improved systolic function
Subpulmonic (right-sided morphologic LV) – Indexed EDV	70 ml/m²	72 ml/m²	Stable
Subpulmonic LV EF	55–60%	58–60%	Preserved

## Discussion

Management of ccTGA in adolescents remains a surgical and clinical challenge because of the complex interplay between morphologic right ventricular dysfunction, associated lesions such as VSD and pulmonary stenosis, and the variable growth of the pulmonary arterial tree. The timing of intervention is crucial, since systemic left-sided morphologic RV failure progresses with age, and late referral often limits surgical options.

In our patient, the decision to proceed with a double-switch operation (atrial and arterial switch) was guided by several factors:

- Systemic left-sided morphologic RV dysfunction: Pre-operative imaging showed a dilated systemic (left-sided morphologic RV) with severely reduced EF (30%). A conservative VSD closure would not have prevented long-term systemic left-sided morphologic RV failure, thus favoring anatomic repair.
- Pulmonary outflow anatomy: Although the pulmonary valve annulus was hypoplastic (Z-score − 3.2) and the McGoon index was borderline (~ 1.3), intraoperative assessment and surgical expertise supported proceeding with a double-switch rather than palliation.
- Alternative surgical options: In such patients, palliative systemic-to-pulmonary shunts (e.g., Blalock–Taussig shunt) or cavo-pulmonary connections (bidirectional Glenn, BDG) are valid alternatives, particularly if pulmonary artery size is inadequate. These were considered but not chosen due to the feasibility of complete repair.

Long-term prognosis for patients undergoing double-switch in adolescence remains guarded, but improved outcomes have been reported when surgical correction is performed before irreversible systemic left-sided morphologic RV dysfunction develops. Lifelong surveillance with echocardiography, CMR, and strain imaging is mandatory to detect late deterioration in LV or RV function, arrhythmias, or outflow tract obstruction.

### Relevance of early imaging

Early diagnosis and timely surgical correction are crucial to improving survival rates and reducing morbidity in patients with ccTGA. Aggressive medical therapy is required on an immediate basis, and surgical interventions must be pursued as soon as feasible to optimize outcomes. This patient survived because of timely intervention, surgical planning and execution leading to improved survival and reduced morbidity.

3D echo can be utilized for better delineation of RV and LV volumes and function.

There are documented instances where individuals with L-type ccTGA remain undiagnosed until their later years, sometimes into their eighth or ninth decades and succumbed to sudden cardiac death [5, 6].

### Limitations

- Single-patient design: a case report involves only single patient, which severely limits the ability to generalize the findings. While this report provides valuable clinical insight, its observations may not be applicable to the broader population of patients with L-type ccTGA. The unique details of this adolescent’s anatomy and management cannot be assumed to predict outcomes in other cases.
- Imaging limitations: Our echocardiographic evaluation was based on two-dimensional measures and Z-scores derived from standard nomograms. It is important to recognize that Z-scores have inherent variability and are not uniformly validated across populations. As one review warns, different Z-score “nomograms” and body-size equations can yield variable results . Thus, the absolute Z-scores reported for RV dimensions may differ if alternative reference data were used, and they should be interpreted cautiously. In addition, we did not employ more advanced imaging techniques that could enhance accuracy. Three-dimensional echocardiography can provide more precise quantification of RV volumes and function, but these were not available in this case. The lack of 3D/quantitative RV assessment is a limitation, as such tools might have better characterized RV remodeling and function than the two-dimensional estimates used here.
- Limited surgical documentation: Finally, the surgical report lacked intraoperative images or detailed quantitative metrics from the operative planning. Having intraoperative imaging (such as epicardial ultrasound or photographs) might have illustrated the anatomy and surgical changes more clearly. Similarly, specific measurements or angles used by the surgeon in correcting the L-loop anatomy were not recorded. This limits the transparency of the procedural rationale. Detailed intraoperative data could have provided additional insight into why particular technical choices were made and how they achieved the postoperative result.

Despite these limitations, this case remains valuable. It documents a rare presentation of L-type ccTGA in an adolescent and the successful management of associated pulmonary stenosis and RV dysfunction. In the absence of large trials for such an uncommon condition, single-case experiences contribute important clinical insights. As noted in the literature, case reports are “the first line of evidence” for novel or complex scenarios. By carefully acknowledging its limitations, this report still adds to the collective understanding of adolescent ccTGA management and highlights areas (such as the need for long-term follow-up and detailed functional assessment) that should be addressed in future cases.

## Conclusion

This adolescent case of L-type ccTGA with pulmonary valve hypoplasia and systemic left-sided morphologic RV dysfunction demonstrated that anatomic “double-switch” repair can yield measurable improvements even in the teenaged patient. By 3 months postoperatively the systemic (morphologic RV) chamber was notably smaller (indexed end-diastolic volume 150→120 mL/m²) and its systolic performance had improved (ejection fraction rising from ~ 30% to 42%). Clinically the patient remained hemodynamically stable, her functional capacity increased from NYHA IV to NYHA II and was able to discontinue inotropic support, with markedly better symptoms and exercise tolerance over the early follow-up period. These encouraging results including successful repair in a 16‑year‑old are in keeping with other reports that anatomic correction can be feasible in select older children. Nevertheless, these findings are drawn from a single case and therefore we cannot emphasize that long-term outcomes and the generalizability of this approach remain uncertain and will require further study and continued follow-up.

This case highlights the value of early suspicion, bedside imaging, and multidisciplinary coordination in managing rare congenital anomalies presenting in acute settings.

## Data Availability

No datasets were generated or analysed during the current study.

## Abbreviations

ARB: Angiotensin receptor blocker
BDG: Bidirectional glenn
BT: Blalock-taussig
CMR: Cardiac magnetic resonance
CT: Computed tomography
ECG: Electrocardiogram
EF: Ejection fraction
LV: Right sided morphologic left ventricle
MPA: Main pulmonary artery
MRI: Magnetic resonance imaging
PA: Pulmonary artery
PV: Pulmonary valve
RV: Left sided morphologic right ventricle
TTE: Transthoracic echocardiography
VSD: Ventricular septal defect

## Declarations

### Patient perspective

The patient’s mother shared that she had been worried for years due to repeated fainting episodes and was relieved to finally receive a diagnosis and see her child alive after such a dreadful condition, also she improved dramatically within a week of admission.
